# A Multi-Attribute Pheromone Ant Secure Routing Algorithm Based on Reputation Value for Sensor Networks

**DOI:** 10.3390/s17030541

**Published:** 2017-03-08

**Authors:** Lin Zhang, Na Yin, Xiong Fu, Qiaomin Lin, Ruchuan Wang

**Affiliations:** 1 College of Computer, Nanjing University of Posts and Telecommunications, Nanjing 210003, China; 15150577400@163.com (N.Y.); fux@njupt.edu.cn (X.F.); wangrc@njupt.edu.cn (R.W.); 2 Jiangsu High Technology Research Key Laboratory for Wireless Sensor Networks, Nanjing 210003, China; lqm@njupt.edu.cn; 3 Institute of Computer Technology, Nanjing University of Posts and Telecommunications, Nanjing 210003, China

**Keywords:** wireless sensor network, MPASR, ant-colony optimization algorithm, trusted secure routing algorithm

## Abstract

With the development of wireless sensor networks, certain network problems have become more prominent, such as limited node resources, low data transmission security, and short network life cycles. To solve these problems effectively, it is important to design an efficient and trusted secure routing algorithm for wireless sensor networks. Traditional ant-colony optimization algorithms exhibit only local convergence, without considering the residual energy of the nodes and many other problems. This paper introduces a multi-attribute pheromone ant secure routing algorithm based on reputation value (MPASR). This algorithm can reduce the energy consumption of a network and improve the reliability of the nodes’ reputations by filtering nodes with higher coincidence rates and improving the method used to update the nodes’ communication behaviors. At the same time, the node reputation value, the residual node energy and the transmission delay are combined to formulate a synthetic pheromone that is used in the formula for calculating the random proportion rule in traditional ant-colony optimization to select the optimal data transmission path. Simulation results show that the improved algorithm can increase both the security of data transmission and the quality of routing service.

## 1. Introduction

A wireless sensor network is composed of a number of sensor nodes. The function of these nodes is to collect data related to certain phenomena in the environment and to transfer these data to a special node called a sink node (a base station or control center). Then, the sink node analyzes the received data in order to make a decision. Wireless sensor networks have recently been widely used in environmental monitoring, medical treatment, and military applications, among others [1,2]. However, because of the unique working environment of wireless sensor networks, they are vulnerable to many security threats, such as Sybil, wormhole and selective forwarding attacks [3]; therefore, the security and credibility of any routing algorithm for wireless sensor networks need to be studied thoroughly. Many researchers have proposed a number of typical routing algorithms for wireless sensor networks, but most of these routing algorithms only consider the limited resources of the wireless sensor network as the primary problem, their design goals are the best route discovery, high energy efficiency and low energy consumption, prolonging the network survival time, and so on, but rarely from the perspective of network security. When there are malicious nodes in the routing process, these algorithms cannot resist most of the attacks. Therefore avoiding the malicious behavior of nodes or the damage possibly caused by aggressive behavior can greatly enhance the security of routing algorithms. In communication networks, trust or reputation can be used to describe the behavior of node communication. The traditional security mechanism, such as, the key management, authentication and encryption technology, cannot effectively solve the security problems caused by the malicious behavior of inner nodes in the network. The security mechanism based on trust has become an effective supplement, which can reduce the security threats to the network. The study of secure routing algorithms based on reputation value for sensor networks is still comparatively rare, and research on routing algorithms for WSNs focuses mainly on protecting the efficiency of data transmission and reducing energy consumption and transmission delay while simultaneously improving the security of the network given limited resources [4,5].

Typical routing protocols can be divided into data-centric, location-based and hierarchical routing protocols. In recent years, many scholars at home and abroad have extensively researched this issue. In [6], Rabelo et al. proposed an approach based on a fuzzy inference system and ant-colony optimization for improving the performance of routing protocols in wireless sensor networks. This algorithm considers energy consumption and proposes a method for estimating route quality by using fuzzy systems to assist the directed diffusion routing protocol, hence increasing the energy efficiency of the network. The directed diffusion protocol has its operation based on the following elements: named data, interest, gradient, and reinforcement. The directed diffusion with fuzzy approach using the ACO algorithm, is more efficient than the others for all metrics, however, shortcomings exist in this algorithm. For example, although the use of a fuzzy inference system adjusted by a trial and error approach makes great use of information to classify the routes, this kind of adjustment is not so powerful as the automatic adjustment allowed by ACO.

In [7], Zeng et al. proposed an improved harmony search (HS)-based energy-efficient routing algorithm for wireless sensor networks. The HS algorithm has a strong global search ability and also has a very simple concept and few parameters. The main steps of the classical HS algorithm are as follows: (1) initialize the optimization problem and algorithm parameters; (2) initialize the harmony memory (HM); (3) improvise a new harmony; (4) update the HM; (5) repeat step (3) and step (4) until the termination criterion is satisfied. This algorithm uses a HS algorithm (a meta-heuristic) to address the WSN routing problem. However, this algorithm does not take network security into consideration. If there are malicious nodes in the routing process, this algorithm cannot resist most of the attacks.

In [8], Jang et al. proposed a distributed energy-balanced unequal clustering routing protocol (DEBUC), which adopts an unequal-clustering mechanism in combination with an inter-cluster multi-hop routing mechanism. The unequal-clustering mechanism organizes the network into clusters of different sizes. By changing the number of nodes in every cluster with respect to the expected relay load, the mechanism maintains more uniform energy consumption among the cluster heads, so that the total energy dissipated for every cluster head is similar. Inter-cluster multi-hop routing mechanism is a method to calculate a route for a transfer of data between a source node and a destination node via at least one intermediate node in a network. By means of a time-based competitive clustering algorithm, DEBUC partitions all nodes into clusters of unequal size, such that clusters closer to the base station are smaller in size. However, DEBUC does not consider multiple concentric layers around the base station, and the effects of event-based networks where the data generation rate at each node is a function of phenomena in the environment.

Almasri et al. proposed a trusted and energy efficient routing protocol for wireless sensor networks (TERP), it is a reliable and energy-efficient routing algorithm based on the destination-sequenced distance-vector protocol (DSDV), which fully accounts for the fact that in a wireless sensor network, the energies of the nodes are limited and the life cycle is short. The DSDV routing algorithm is based on the idea of the classical Bellman-Ford routing algorithm with certain improvements. Every mobile station maintains a routing table that lists all available destinations, the number of hops to reach the destination and the sequence number assigned by the destination node. The sequence number is used to distinguish stale routes from new ones to avoid the formation of loops. The stations periodically transmit their routing tables to their immediate neighbors. TERP considers the security issues that arise during the process of passing data from one node to another and is consequently able to greatly improve the security of data communication. However, the result shows that TERP takes longer time than DSDV because of the amount of information sent to each node, which may increase the delay jitter and have a certain impact on the network [9].

Cui et al. applied ant-colony optimization to solve the routing problem, considering the real-time performance requirements and the limited energy of the wireless sensor network. Their algorithm limits the scope of the search for the next node based on the search angle and uses a direction-based pheromone designed to guide the ant colony to reach the destination node. However, the WSN routing protocols is vulnerable, which has not be designed and realized routing protocol with security mechanism [10].

To balance the load and prolong the lifetime of a wireless sensor network, Xie et al. proposed a clustering routing protocol, CRT2FLACO, based on fuzzy logic and ant-colony optimization. The residual energy, the number of neighboring nodes and the distance to the base station for each node are considered in the fuzzy logic to balance the load; in addition, the cluster heads are linked into a chain using the ACO algorithm to reduce transmission energy consumption. The CRT2FLACO protocol can effectively balance the network load and save energy so as to prolong the network lifetime. However, the rules in this protocol are fixed and defined by experience [11].

There exist some shortcomings of the papers described above. For example, the complexity of the algorithm may be very high, the security of the nodes during communication may not be taken into account, the convergence rate may be slow, which may make the algorithm prone to stagnation, or the energy consumption of the nodes may not be considered. To address these problems, this paper proposes a multi-attribute pheromone ant secure routing algorithm based on reputation value (MPASR). The algorithm considers the safety concerns for the nodes during the process of transmitting data. Moreover, the concept of a node's reputation value is introduced, and the node reputation values, the residual node energies, and the transmission delay are combined to define the pheromone used in ant-colony optimization to improve communication security, protect the network’s life cycle, and ultimately provide a better routing service. The main contributions of this paper are as follows:

(1) To improve network security, this paper introduces node reputation values into the routing algorithm for a wireless sensor network. The reputation value of a node is composed of a direct credit value and an indirect credit value. In the calculation of the indirect credit value, nodes with higher coincidence rates are removed via a filtering mechanism to avoid unnecessarily repeated calculations. The node reputation values are introduced to defend against internal threats to the wireless sensor network, which improves the security of the node communication process and the communication quality and thereby, in turn, increases the network’s resistance to external threats.
(2) This paper proposes an improved ant-colony optimization algorithm for wireless sensor networks. An ant-colony optimization algorithm is a meta-heuristic algorithm for combinatorial optimization problems; it is suitable for the selection of node paths and has good potential for application in wireless sensor networks. In the improved ant-colony optimization algorithm, the node reputation value, the residual node energy, and the transmission delay are combined to formulate a synthetic pheromone, and full consideration is given to the security of data transmission while balancing this concern with the energy consumption of the network nodes to avoid the premature death of some nodes.

The organizational structure of this article is as follows: the second section introduces the concepts related to trusted routing algorithms. In the third section, we propose the MPASR trusted secure routing algorithm for WSNs, and the fourth section presents the results of a simulation experiment. Finally, the fifth section provides a summary of this article.

## 2. Related Concepts

With the continuous development of science and technology, human beings are paying increasing attention to security issues. Security can be classified into two main aspects: privacy protection and trust. Although scholars at home and abroad have studied security in various fields, a truly high degree of security has not yet been achieved [12,13,14,15]. Currently, many scholars are studying wireless sensor network routing algorithms, mostly in an attempt to solve the problem of limited node energy, however, they rarely consider the security issues posed by malicious nodes, and much less consider safety and energy issues simultaneously. Therefore, in this paper, to improve the energy efficiency of wireless sensor networks, a suitable trust mechanism is introduced into the routing protocol. In a practical sense, trust can be simply defined as the confidence that the nodes will behave as expected. There are two main types of trust measurements: direct and indirect [16]. To measure the trust between nodes more directly, this paper introduces the concept of the reputation value of a node and uses this variable to determine whether the node is credible or malicious; the definition of a node’s reputation value is given below.

**Definition 1** (Node Reputation Value)**.** *The reputation value of a node is defined as an absolute value for a specific period and environment, which is calculated using an appropriate evaluation model of the node’s credibility based on a synthetic analysis of the node’s historical behavior*.

It represents a comprehensive image of the node within the set of all nodes and is a summary of the overall evaluation results. Ants have features of self-organization, self-adaption, mutual communication and collaboration. Ant-colony optimization simulates their intelligent behavior and can solve many complex problem. Ant-colony optimization is proposed as a new type of simulated evolutionary algorithm in the early 1990s, which is also a very important method of cluster analysis. It has strong extensibility and robustness and can adapt to the dynamic environment, so it is suitable for the design of dynamic wireless sensor networks. Ant-colony optimization is widely applied in ad hoc networks, wireless sensor networks, etc., because of its characteristics of self-organization, self-optimization, and dynamic topology [17]. The underlying concept of ant-colony optimization is described in Definition 2. Traditional ant-colony optimization algorithms do not consider the unique characteristics of wireless sensor networks. Therefore, it is necessary to improve upon the traditional ant-colony optimization algorithms for this purpose. We integrate the reputation values, energies and time delays of the nodes into the algorithm to increase its effectiveness in solving problems involving wireless sensor networks.

**Definition 2** (Ant-Colony Optimization)**.** *Ant-colony algorithms are swarm-intelligence optimization algorithms inspired by research on the behavior of ant colonies in the real world that has revealed that ants are capable of finding the shortest path between a food source and their nest*.

Ants leave pheromones along each path, and the amount of pheromones on a path indicates the number of ants that have walked along that path; the more ants that have walked along a path, the closer that path is to food. Eventually, all ants will choose the path with the most pheromones, which is the shortest path between the nest and food [18]. Ants leave pheromone on each path, the more pheromones show that more ants are on the path, and the closer the food is to the path. In the end, all ants will choose the path left by the most pheromones, which is the shortest path between the nest and the food. In our approach, we use the Ant System algorithm, which is the most basic ant colony optimization algorithm. The algorithm is simple and easy to understand, which has the advantage of robust, excellent distributed calculated mechanism, easy to combine with other methods.

## 3. Ant Colony Optimization

**Figure 1:**
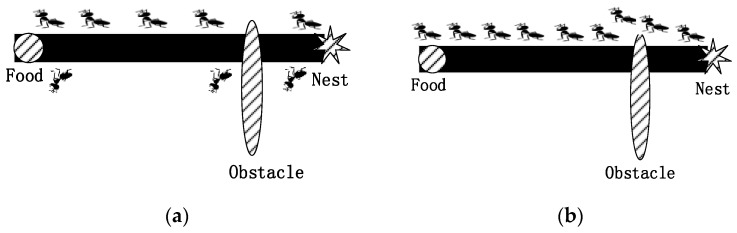
Ants find the shortest path. (**a**) The initial state of selecting path; (**b**) the final state of selecting path.

As shown in Figure 1a, when ants finding food deposit pheromones on the way to their nests, the other ants then follow these deposited pheromones. In this way, ants can search for the shortest path from their nest to food with the pheromone information.

The ant colony optimization algorithm (ACO) was first used to solve the traveling salesman problem (TSP), which is the problem of finding the shortest path that traverses all cities exactly once and then returning to the starting city. In the ACO algorithm for TSP, the number of cities is *N*, and there are *M* ants. Each ant chooses the next city with a probability in accordance with the distance between the current city and the next one and in accordance with pheromone information. The state transition rule from city *i* to *j* for the *k*-th ant is as follows:

(1) $$Z_{ij}^{k}(t)=\left\{ \begin{array}{l} \frac{{\left[P_{ij}(t)\right]}^{\alpha }\times {\left[D_{ij}\right]}^{\beta }}{\sum_{k\in allowed_{k}}{\left[P_{ij}(t)\right]}^{\alpha }\times {\left[D_{ij}\right]}^{\beta }},\mathrm{if}\;j\subset allowed_{k}\\ 0,others \end{array}\right.$$

where *allowed_k_* is the set of remainder cities, *α* and *β* are two constants, which determine the relative influence of the pheromone and the heuristic on the decision respectively. *D_ij_* is the heuristic information, and *D_ij_* = 1/*d_ij_*, where *d_ij_* is the distance between *i* and *j* and *p_ij_*(*t*) is the integrated pheromone concentration. The updated value *p_ij_*(*t*)’ is as follows:

(2) $$p_{ij}(t)\prime =(1-\delta )p_{ij}(t)+\delta \Delta p_{ij}(t)$$

where *δ* is the pheromone evaporation rate. The pheromone field is updated after *M* ants finish the *N* cities of travel. Δ*p_ij_*(*t*) is the pheromone increment associated with ants traveling on the path from *i* to *j*:

(3) $$\Delta p_{ij}\left(t\right)=\sum_{k=1}^{m}\Delta p_{ij}^{k}\left(t\right)$$

$\Delta p_{ij}^{k}\left(t\right)$ is the pheromone increment of ant *k*. *Q* is the pheromone strength, which is a constant.

(4) $$\Delta p_{ij}^{k}\left(t\right)=\left\{ \begin{array}{l} Q,\;\mathrm{if}\;j\in \;\mathrm{solution}\;\mathrm{of}\;\mathrm{ant}\;k\\ 0,\;\mathrm{otherwise}. \end{array}\right.$$

This iteration process goes on until termination conditions is satisfied. For example, when a certain number of iterations have been achieved or when the best path does not change after several iterations, this process will stop.

## 4. Trusted Network Secure Routing Algorithm

The main goals of the secure routing algorithm proposed in this paper are to reduce the energy consumption of wireless sensor networks, optimize the existing secure routing protocols for such networks, find a suitable path for communication on the premise that the node conditions are satisfied, and achieve high security performance.

**Figure 2:**
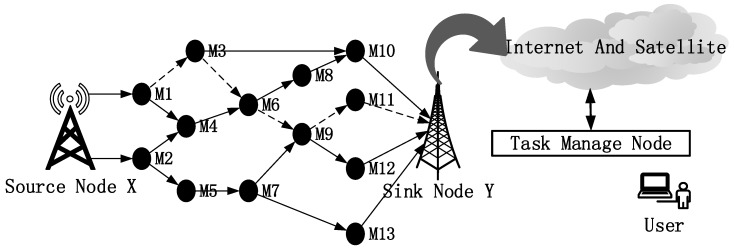
Data transmission path for communication in a wireless sensor network.

The source node for communication is denoted by *X*, and the destination node is denoted by *Y*. To simplify the analysis process, it is assumed that the source node is always ready to send data, without being subject to influencing factors. The data transfer process is illustrated in Figure 2. The problem to be solved in this paper can be abstracted as the selection of an optimal path from *X* to *Y* based on reputation and ant-colony optimization, as indicated by the dotted line in the diagram.

### 4.1. Node Reputation

Trust is a very complicated concept that has been studied for almost a century. Trust is a social attribute in human society and is generally regarded as an intuitive concept. It is subjective and can be established between two individuals in an environment to ensure relative safety. A wireless sensor network can be seen as a microcosm of human society, and the trust relationship between nodes can be expressed in terms of node reputation values. In a wireless sensor network, communication mainly relies on the mutual cooperation between nodes; because the network does not have a fixed topology, establishing good trust relationships by introducing the node reputation mechanism into a wireless sensor network can effectively improve the network’s security performance against internal threats.

In research on trust values, the most commonly used type of trust value is one based on the Beta distribution, which is simple and flexible and is well suited for computing values to represent the communication reputations of nodes. The probability density function of the Beta distribution *beta*(*α,β*) is shown in Equation (5):

(5) $$f\left(x|\alpha ,\beta \right)=\frac{\Gamma \left(\alpha +\beta \right)}{\Gamma \left(\alpha \right)\Gamma \left(\beta \right)}x^{\alpha -1}{\left(1-x\right)}^{\beta -1}$$

In this formula, *x* should satisfy 0 ≤ *x* ≤ 1; moreover, *a* > 0 and *b* > 0. The BRSN reputation-evaluation model uses the Beta distribution to calculate the reputation value of a node [19,20]:

(6) $$R_{ij}=\frac{\alpha_{ij}+1}{\alpha_{ij}+\beta_{ij}+2}$$

where *R_ij_* represents the reputation-evaluation value of node *i* with respect to node *j*, *α_ij_* and *β_ij_* denote the number of successful communication behaviors and the number of failed communication behaviors, respectively.

In this paper, the method of calculating the node reputation value in the DPMA-MD algorithm [21] is improved. In the calculation of the reputation values, the DPMA-MD algorithm uses the concepts of direct credit evaluation and indirect credit evaluation, and the two corresponding types of reputation values are integrated to obtain the final reputation value for a node. However, this method only considers how to calculate the node reputation value; it does not consider the effective utilization of energy, the complexity of the calculation, etc. Therefore, in this paper, we propose a new algorithm for calculating the reputation value of a node.

**Figure 3:**
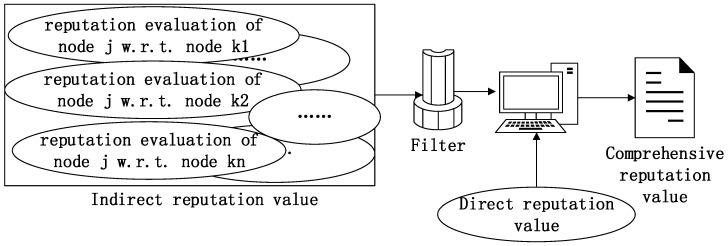
The framework for calculating node reputation values.

The nodes in a wireless sensor network are randomly distributed. The reputation-evaluation information is very similar for some nodes, and if their sensing areas intersect, then the calculation of the credit evaluations of their information will involve duplicate computations, thereby increasing the computational complexity of the algorithm and resulting in unnecessary energy consumption [22]. Therefore, this paper introduces a filtering mechanism into the calculation of the indirect reputation value, which reduces the repetition of computations and eliminates the influence of repeated information on the objective situation. The process is illustrated in Figure 3.

#### 4.1.1. Indirect Reputation Value

**Figure 4:**
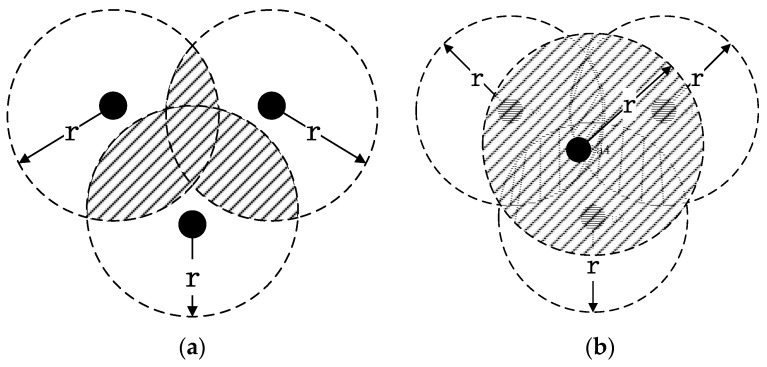
Overlap among deployed nodes. (**a**) Three nodes cover the entire area; (**b**) four nodes cover the entire area.

Wireless sensor networks show high randomness in the deployment of nodes; there will thus be large overlaps in the perceptual ranges of the nodes. As shown in the figure below, in Figure 4a, the three nodes cover the entire area, whereas in Figure 4b, the scope of the fourth node is completely coincident with those of the other nodes. If this node is filtered out, then the energy consumption of the network can be reduced without affecting the node credibility evaluation.

To filter out such nodes, this paper introduces the concept of the coincidence rate to measure the contact ratio of information between nodes. The nodes are grouped according to a certain distance. If the number of nodes in a group is 1, there is no need to filter; otherwise, the coincidence rate of each packet is calculated.

The joint information entropy can be used to characterize the relationships among multiple data sources, but its calculation formula is difficult to realize. Therefore, this paper adopts the method for the approximate calculation of the relative joint information entropy between information sources presented in [23]. Sundeep and others believe that the distance between two information sources can be used to measure the degree of correlation between those sources; the specific formula is as follows:

(7) $$H_{n}\left(\overset{-}{d}\right)=H_{1}+\left(n-1\right)\left[1-\frac{1}{\left(\frac{\overset{-}{d}}{c}+1\right)}\right]H_{1}$$

where $H_{n}\left(\bar{d}\right)$ vrepresents the approximate joint information entropy among *n* sources, $\bar{d}$ represents the average distance between the sources, and *c* is a constant that reflects the degree of spatial correlation among the information sources.

From the above formula, we can obtain the coincidence rate $D_{n}\left(\bar{d}\right)$, which is defined as the approximate joint information entropy $H_{n}\left(\bar{d}\right)$ divided by the node information entropy as a standard reference:

(8) $$D_{n}\left(\overset{-}{d}\right)=1+\left(n-1\right)\left[1-\frac{1}{\left(\frac{\overset{-}{d}}{c}+1\right)}\right]$$

Finally, when the coincidence rate among the nodes is lower than a specified coincidence rate threshold, the group retains only those nodes with the maximum residual energies, and all other nodes are filtered out. This threshold should be set in accordance with the size of the network. After filtering, the indirect credit value parameters of the remaining packet nodes are determined based on the Jøsang trust principle [24]:

(9) $$\left\{ \begin{array}{l} \alpha_{ij\_in}^{k}=\frac{2\alpha_{ik}\alpha_{kj}}{\left(\beta_{ik}+2\right)(\alpha_{kj}+\beta_{kj}+2)+2\alpha_{ik}}\\ \beta_{ij\_in}^{k}=\frac{2\alpha_{ik}\beta_{kj}}{\left(\beta_{ik}+2\right)(\alpha_{kj}+\beta_{kj}+2)+2\alpha_{ik}} \end{array}\right.$$

where $\alpha_{ij\_in}^{k}$ ($\beta_{ij\_in}^{k}$) indicates that node *i* obtains the normal (abnormal) communication times of nodes *j* from other nodes, and *α_ik_* (*β_ik_*) indicates that node *i* obtains normal (abnormal) communication times of nodes *k*. Equation (9) can be substituted into Equation (6) to obtain the indirect reputation value *R_ij_in_* of the node.

#### 4.1.2. Communication-Behavior Updating

In [15], node communication behavior is updated by simply adding 1, but a malicious node may initially exhibit normal communication behavior to obtain a high reputation value, or initially normal nodes may later be captured by an attacking node; thus, normal early statistics may mask the abnormal behavior of such nodes in a later period. To avoid this situation, this paper introduces the weakening factor *η*, which takes values in the range between 0 and 1 and can effectively weaken the statistical information from earlier times. Under the assumption that the update cycle of the network system has a period of Δ*T*, the normal and abnormal communication behaviors are updated as follows:

(10) $$\left\{ \begin{array}{l} \alpha_{ij}=\eta \cdot \alpha_{ij}+\alpha_{ij}(\Delta T)\\ \beta_{ij}=\eta \cdot \beta_{ij}+\beta_{ij}(\Delta T) \end{array}\right.$$

#### 4.1.3. Comprehensive Reputation Value

By combining the results from the sections above, the final formula for the reputation value of a node is obtained as follows:

(11) $$R_{ij}=\lambda R_{ij-d}+(1-\lambda )R_{ij-in}$$

In Equation (11), *R_ij_d_* represents the direct reputation-evaluation value of node *i* with respect to node *j*, *R_ij_in_* represents the indirect reputation value of node *i* with respect to node *j* obtained through node *k*, and *λ* represents the weight of the direct credit value; the greater the value of *λ* is, the greater will be the influence of the direct credit value on the total credit value. This paper assumes that all nodes in the sensor network have the ability to detect the communication abilities of other nodes. Node *i* has direct trust relationships with multiple nodes and can accept the recommendations of adjacent nodes for trusted nodes *j*. Trust is based on observations and evaluations of other nodes collected over a period of time to evaluate the node credibility.

### 4.2. Residual Energy

In a wireless sensor network, the power of some nodes on a path may be greatly reduced because of excessive use of that path, and these nodes will then fail because of their low residual energy. Therefore, the routing algorithm must consider the remaining energy levels of the nodes. In this paper, the energy consumptions of the nodes are calculated using the wireless communication model presented in [25]. Two communication models are considered in this paper: a free-space model and a multi-path attenuation model. When the communication distance is greater than *d*_0_, the multi-path attenuation model is adopted; otherwise, the free-space model is adopted. The initial energy of a node is denoted by *E_i_*, and the residual energy of the node at time *t* is denoted by *E_re_*; the residual energy can be obtained using the following equations:

(12) $$E_{re}=E_{i}-E_{c}\left(t\right)$$

(13) $$E_{c}\left(t\right)=E_{tx}+E_{rx}$$

(14) $$E_{tx}=\left\{ \begin{array}{l} L*E_{ele}+L*\varepsilon_{fs}*d^{2},d<d_{0}\\ L*E_{ele}+L*\varepsilon_{mp}*d^{4},d\geq d_{0} \end{array}\right.$$

(15) $$E_{rx}=L*E_{ele}$$

In these formulas, *E_tx_* is the total transmission energy consumption of the sender, *E_rx_* is the total energy consumption of the receiver, *E_ele_* represents the energy consumed for the transmission and reception of data over a wireless transmission line, and *ε_fs_* and *ε_mp_* are the energy consumption parameters for free-space and multi-path communication, respectively. *L* is the size of the transmitted data, and *d* is the distance between the sender and receiver.

### 4.3. Communication Delay

The routing protocol used in a wireless sensor network has a close relationship with the communication delay: the longer the delay is, the greater the impact on the network’s normal communication will be. In this paper, the distance between nodes is considered to represent the communication delay *T_ij_*. One might simply use the physical distance between two nodes to represent their communication delay; however, a situation can arise in which the physical distance between the two nodes is very short but the node voltage is close to the minimum voltage value of the sensor, causing the communication ability of the nodes to be very poor. Thus, in this case, the effective distance between the two nodes is very far. Therefore, in this paper, we propose the following concept of the effective distance to provide a more reasonable representation of the communication delay:

(16) $$T_{ij}={\left[\frac{1}{V_{0}-V_{\min}}\times (d_{ij}){}^{2}\right]}^{-1}$$

where *V*_0_ is the current operating voltage of the sensor, which is initialized at 3 V and, when the system is running, is equal to the voltage value of the node in the wireless sensor network; *V*_min_ is a constant that specifies a critical voltage and is generally set to 2.7 V; and *d_ij_* represents the Manhattan metric distance between node *i* and node *j*, which is defined in Equation (17). The above formula considers not only the relationship between the energy and the distance between the nodes but also the change in voltage. This formula causes the effective distance between the nodes to change very quickly when the voltage is near the minimum value, which makes the formula very useful.

Manhattan metric: Suppose that *i* = (*x_i_*_1_,*x_i_*_2_,…,*x_ip_*) and *j* = (*x_j_*_1_,*x_j_*_2_,…,*x_jp_*) are two objects described by *P* numerical attributes. The definition of the Manhattan metric between objects *i* and *j* is shown below:

(17) $$d(i,j)=\left|x_{i1}-x_{j1}\right|+\cdots +\left|x_{ip}-x_{jp}\right|$$

### 4.4. Reputation and Ant-Colony Optimization

From Definition 2 in the second section, we know that ant-colony optimization can be used to solve an optimal path problem, which is consistent with the goal of a secure routing protocol for wireless sensor networks. Therefore, in this paper, the improved ant-colony optimization algorithm is applied in the routing protocol to select the optimal path for the network to use for data transmission.

#### 4.4.1. Pheromone

An ant-colony optimization algorithm dynamically adjusts the pheromone concentration on each path to achieve the purpose of selecting the current optimal path [26]. Most traditional ant-colony optimization algorithms are based on a single attribute. Although this approach simplifies the algorithm, it tends to cause other important attributes of the nodes to be ignored, which is not conducive to energy balance and has some effect on the experimental results. Therefore, in this paper, we adopt a multi-attribute pheromone that includes the reputation value as the main basis on which to select the optimal path for data transmission [27,28].

In this paper, we combine the reputation value *R_ij_*, the residual energy *E_ij_* and the communication delay *T_ij_* to formulate a pheromone *P_ij_* that considers trust, energy, time delay and other factors. This approach not only improves the safety of the network but also avoids excessive energy consumption, which would otherwise result in the premature deaths of individual nodes. As shown in Equation (14), the approach proposed in this paper does not simply sum the values of the attributes. Instead, the calculation is based on the particular relationship between each attribute and the pheromone value: if they are directly proportional, then the attribute is directly multiplied by a weighting factor *θ* and included in the sum, whereas if the attribute is inversely proportional to the pheromone, then the inverse of the attribute is multiplied by the corresponding weighting factor and included in the sum. This approach ensures that all attribute terms are ultimately kept in direct proportion to the pheromone:

(18) $$P_{ij}=\theta_{1}R_{ij}+\theta_{2}E_{ij}+\theta_{3}{\left(T_{ij}\right)}^{-1}$$

In the formula above, *θ*_1_ + *θ*_2_ + *θ*_3_ = 1. The weights of the three factors must be selected to determine the degrees to which security, energy, and delay are considered in the choice of the next-hop node. The node reputation values, residual energies and time delays are obtained as described in Section 4.1, Section 4.2 and Section 4.3.

#### 4.4.2. Path Construction

The proposed routing protocol for wireless sensor networks is based on the comprehensive pheromone values of the nodes. The potential and optimized next-hop nodes are determined in accordance with the state transition rule. Under normal circumstances, the probability that the node with the largest comprehensive pheromone value will be selected is relatively large. In the algorithm, we use a probability roulette wheel mechanism to select the following node at a point. The specific formula for the synthetic pheromone is given as Equation (18) in Section 4.4.1. Based on this expression, the formula for calculating the state transition rule [29,30,31] is shown in Equation (1).

- *i* and *j* are nodes in the network.
- *P_ij_*(*t*) is the integrated pheromone concentration on the path from *i* to *j* at time *t*.
- *allowed_k_* represents the set of valid next-hop nodes that have not yet been visited.
- *α* and *β* are two constants. They are the weight values for the synthetic pheromone and the heuristic information, respectively, and they satisfy *α* ≥ 0 and *β* ≥ 0. *α* reflects the degree of influence of the comprehensive pheromone on the path selection, and *β* represents the effect of the heuristic information on the path selection. Increasing the value of *α* will increase the influence of the pheromone in the path selection process. The pheromone used in the improved algorithm presented in this paper is based on many factors, and its role should be emphasized; thus, in this paper, *α* = 2 and *β* = 1.
- *D_ij_* is the heuristic information. The traditional heuristic information is the reciprocal of the distance between two nodes. However, in the MPASR algorithm, the main emphasis is placed on the influences of the node reputation, residual energy and transmission delay on data packet transmission. Thus, the weight value for the heuristic information is set to 1 in this paper.

#### 4.4.3. Integrated Pheromone Updating

The ants will leave more information elements along shorter paths. To simulate this phenomenon, when each ant reaches the end of its path, the pheromone concentration on that path must be updated. The amount of pheromone on a path will increase as the number of ants that have traveled along it increases, and the pheromone will also evaporate as time elapses. Therefore, we consider both factors in the process of pheromone updating, which is shown in Equation (2). Where *δ* is the pheromone evaporation rate. Both ant-colony algorithms and genetic algorithms, as well as a variety of other simulated evolutionary algorithms, exhibit slow convergence rates and can easily fall into local optima. The pheromone evaporation rate is directly related to the global search capability of the ant-colony algorithm and its convergence rate: if the value is small, it may cause residual pheromones to dissipate too quickly, which is not conducive to pheromone accumulation, whereas if the value *δ* is too large, then the algorithm may have a high risk of falling into a locally optimal solution. Previous studies in the literature indicate that an evaporation rate between 0.5 and 0.9 is best, therefore, the evaporation rate is set to 0.6 in the proposed algorithm. Δ*p_ij_*(*t*) is the pheromone increment associated with ants traveling on the path from *i* to *j*, and its value is fixed, which can be achieved by Equations (3) and (4). Among them, $\Delta p_{ij}^{k}\left(t\right)$ is the pheromone increment associated with ant *k* traveling on the path from *i* to *j*. *Q* is the pheromone strength, which can affect the convergence speed of algorithm to a certain extent. And previous studies in the literature indicate that *Q* = 100 is suitable.

The updating formula for the synthetic pheromone is only used during the path optimization process; when a new path query begins, the initial amounts of pheromone on all edges must be recalculated according to the node attribute information at that time.

### 4.5. The Specific Process of the MPASR Algorithm

**Figure 5:**
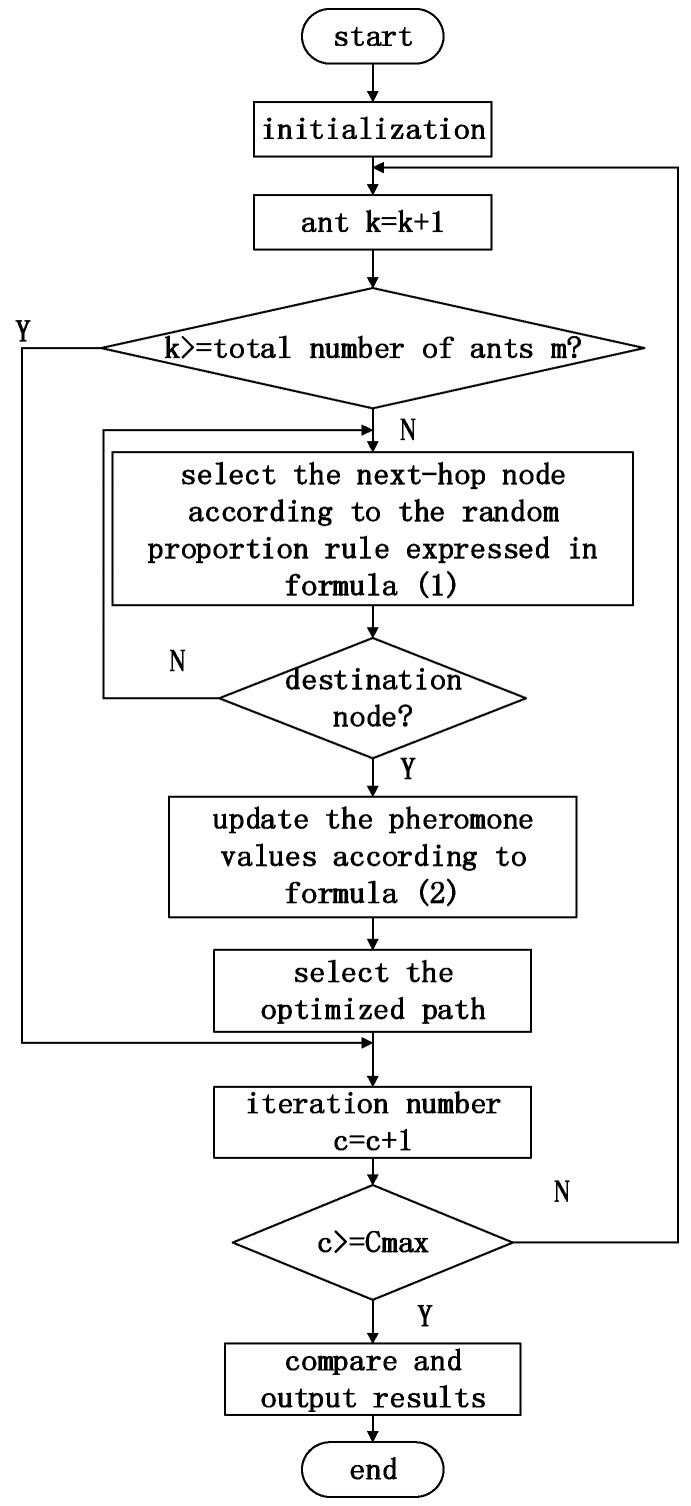
Flow chart of the proposed wireless sensor network algorithm based on ant-colony reputation values.

For the calculation of the reputation values and the application of ant-colony optimization, it is necessary to release many ants onto each path to record the individual attribute information for each node. Then, the optimal path is selected based on the pheromone values. The specific algorithm for implementing this process is shown below, and the specific flow chart is shown in Figure 5.

Step 1: Initialize the parameters. The maximum number of iterations is *C_max_*. Use the variable *c* to record the number of iterations performed during the execution of the algorithm; its initial value is 1. *m* ants will eventually be randomly placed at the *n* sensor nodes. Use the variable *k* to record the cumulative number of ants; its initial value is 0.
Step 2: Increase the number of ants: *k* = *k* + 1. When *k* > *m*, begin a new round of path selection.
Step 3: Based on the node attribute information (node reputation value, residual energy and transmission delay), calculate the pheromone value and insert it into Equation (1) to calculate the random proportion rule to be used to select the next-hop node.
Step 4: After selecting the next-hop node, record the corresponding node attribute information needed to calculate the comprehensive pheromone value. If the next-hop node is the destination node, then terminate the query process and begin the traversal of the next ant; otherwise, continue to choose the next-hop node as described in Step 3.
Step 5: According to the pheromone update Equation (2), update the amount of pheromone on the path, and return to Step 2.
Step 6: For each sensor node, calculate the credibility, the path length, and the remaining energy, and select the optimal path after the current iteration.
Step 7: Update the number of iterations: *c* = *c* + 1. If *c* > *C_max_*, go directly to Step 8 and terminate the algorithm; otherwise, go to Step 2 to start a new round of path selection.
Step 8: Compare the paths selected over all rounds and select the optimal one as the output. The algorithm ends.

## 5. Simulation Experiment and Analysis

In this section, we report the use of the MATLAB software suite to program, simulate, and validate of the MPASR trusted routing algorithm. For the simulation environment, the operating system Windows 8 and the compiler software MATLAB 7.0.1 were used; the processor was an AMD A4-3300M APU (AMD, Cicero, IL, USA) equipped with a Radeon™ 1.90 GHz HD graphics card (Intel, Amsterdam, NY, USA) and 4 GB of memory. To more intuitively illustrate the improvement in security performance achieved by the algorithm proposed in this paper, we compared it with implementations of routing using an Ant-Colony Optimization Router Chip (ACORC) [32], QOS-Particle Swarm Optimization (QOS-PSO) [33], and the Throughput-Aware Novel ACO-Based Routing Protocol (TANARP) [34]. These three routing algorithms were also applied in the simulations, and their results were compared with those of the algorithm proposed in this paper.

The ACORC algorithm, proposed by Okdem and Karaboga, is a multi-path routing protocol. Upon the failure of a node, the algorithm can provide reliable communication. More importantly, the data packets to be sent do not need to retain information on the nodes that have been visited, which reduces the size of the data packets and consequently conserves node energy. This routing algorithm based on ant-colony optimization considers only the path length in the pheromone computations. The QOS-PSO algorithm treats the integrated service quality of the wireless sensor network as the adaptive value considered in the particle swarm optimization algorithm, thereby improving the overall network performance, supporting the dynamic nature of the wireless sensor network, and ensuring the quality of service of wireless sensor network applications. Because of energy limitations, it is very difficult to design a reasonable and efficient routing protocol for wireless sensor networks. The TANARP algorithm primarily uses ant-colony optimization algorithm to select the optimal path; the ant-colony algorithm considers the delay, energy and frequency of each node, along with other factors. The TANARP algorithm was developed by applying PA-SHORT to the NARP algorithm [35]. It improves the throughput performance of a wireless sensor network by allowing some node mobility.

**Figure 6:**
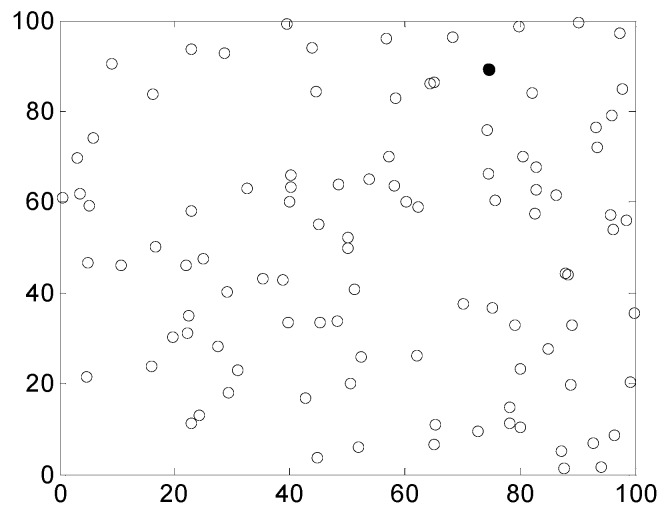
Diagram of the node deployment in the wireless sensor network used in the simulation experiments.

In the simulation experiments, the performances of these four types of trusted routing algorithms were compared under the same conditions. The packet loss rate, packet delivery ratio and average delay were compared. In each experiment, 100 nodes of a wireless sensor network were randomly distributed in a square area of 100 m × 100 m and were provided with a sink node to form a wireless sensor network environment with a planar topology, as shown in Figure 6, the black dot in Figure 6 represents the sink node.

The specific parameters of the simulated network environment used for network deployment are shown in Table 1.

In order to analyze the security of routing algorithm, the complex network is simplified, and the network nodes are divided into two kinds: normal and malicious nodes. Meanwhile, the reputation value of a node is known, because the higher the reputation value of the node is, the more credible the node is. The reputation value of the normal and malicious nodes is 0.8 and 0.3 respectively. According to Section 4.1.2, the weakening factor *η* takes values in the range between 0 and 1. Here, the weakening factor is introduced to effectively weaken the statistical information from earlier times, so the value of the factor should be smaller (0.4). From the above Equation (3), we can obtain the coincidence rate $D_{n}\left(\bar{d}\right)$. Table 2 shows the number of redundant nodes, which have been removed under different parameter settings.

It can be seen that if the node coincidence threshold is set to 1, any redundant nodes are not filtered out. With the increasing of threshold value, the filtering effect of redundant information is more obvious, especially when the node group radius is 10 m. If the node group radius is larger, the number of nodes will increase, the coincidence rate will be higher than threshold, so that the redundant nodes cannot be filtered out. If the node group radius is smaller, the number of nodes in a group will be 1, there is no need to filter. So in the simulation experiment, node group radius is 10 m, and node coincidence threshold is 1.75.

### 5.1. Scenario One

In this experiment, a fixed number of packets were sent to the sink node from a fixed data source node. The source node and the sink node were located at opposite ends of the network. The packet delivery ratios and average energy consumptions of the proposed algorithm, the TANARP algorithm, the ACORC algorithm and the QOS-PSO algorithm were compared for different numbers of malicious nodes. The packet delivery ratio is defined as the ratio of the number of data packets delivered to the total number of packets sent from the source node to the sink node.

**Figure 7:**
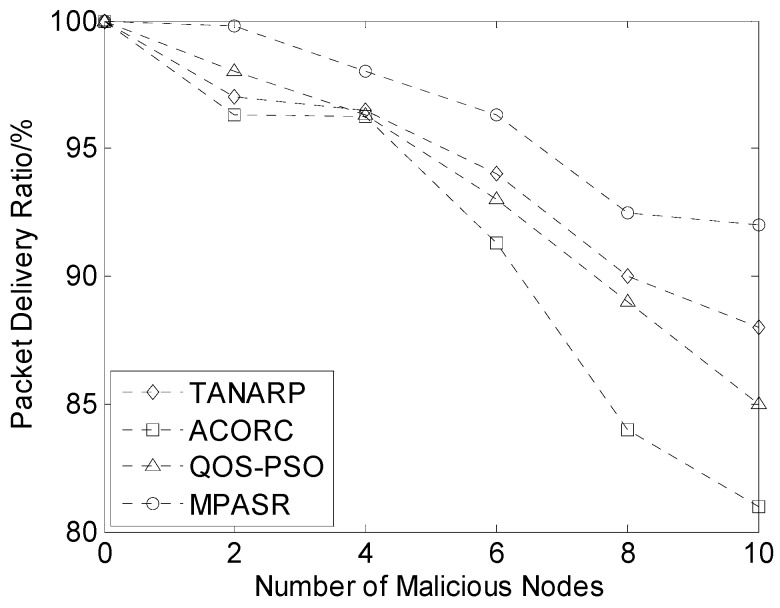
Comparison of packet delivery ratios for different numbers of malicious nodes.

The final results of the experiment are shown in Figure 7. When no malicious node exists in the network, the proposed algorithm, TANARP, ACORC and QOS-PSO all present similar results. It is obvious that as the number of malicious nodes increases, the packet delivery ratios of the three algorithms exhibit a decreasing trend. Of the three algorithms, the rate of decrease of ACORC’s delivery ratio is the largest, that of the algorithm proposed in this paper is the smallest, and that of the QOS-POS algorithm is in the middle. The main reason for this phenomenon is that the algorithm proposed in this paper not only considers factors such as time delay and residual energy in the pheromone calculation process but also treats the reputation value as a component of the pheromone. When selecting the next-hop node, the current node considers the node reputation values and will jump over a node with a lower reputation value. The security factor of the node reputation is used to select the transmission path; therefore, this algorithm can function well even in the presence of a large number of malicious nodes while also maintaining a high rate of packet delivery, thereby effectively reducing the damage to data transmission caused by malicious nodes. Since the ACORC algorithm considers only the path length in pheromone calculation, the robustness of the nodes during the transmission process is relatively poor.

**Figure 8:**
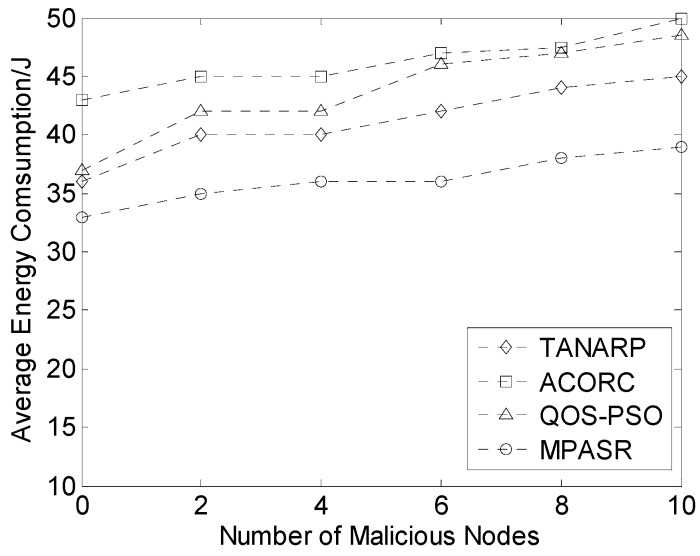
Comparison of the average energy consumptions for different numbers of malicious nodes.

Figure 8 compares the average energy consumptions of the four algorithms with respect to the number of malicious nodes. As seen from the plot, the average energy consumption of the proposed algorithm is lower than those of the other three algorithms. When no malicious node exists in the network, the average energy consumption of the proposed algorithm, TANARP and QOS-PSO are very close, because they all have considered energy efficiency and energy consumption balance, especially for small sized networks. However, the ACORC algorithm based on ant-colony optimization considers only the path length in the pheromone computations, so its average energy consumption is relatively large. As the number of malicious nodes increases, the energy consumptions of all four algorithms increase. The other three algorithms do not fully consider network security factors in their routing protocols, whereas the proposed algorithm measures the node credibility by monitoring the communication behaviors of the nodes. Additionally, in the credibility calculation, some invalid nodes are excluded through filtering, which greatly reduces the energy consumption of the network. Therefore, the energy consumption of the proposed algorithm is the lowest. As the number of malicious nodes increases, the MPASR algorithm uses its safety factor (node reputation) to effectively avoid malicious nodes, thereby reducing the influence of these nodes on the network and allowing the energy consumption of the nodes to remain relatively stable.

### 5.2. Scenario Two

**Figure 9:**
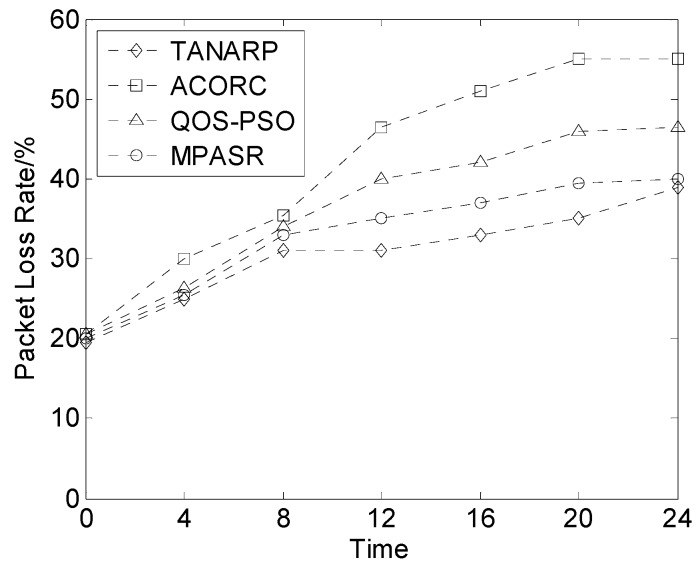
Comparison of packet loss rates over time.

In this experiment, the packet loss rates of the four algorithms were compared. The ratio of the number of data packets lost during the data transmission to the total number of packets sent by the source node is the data packet loss rate. Scenario two is different from scenario one in that the quantity considered for comparison is measured as a function of time. From Figure 9, we can intuitively see that the four algorithms’ packet loss rates are very close for the first 8 s, but as time passes, the packet loss rates of QOS-POS and ACORC become higher than that of the algorithm proposed in this paper. The rate for the TANARP algorithm is lower than that for the proposed algorithm, but the difference is very small, and the packet loss rates of all four algorithms increase over time. Eventually, the packet loss rates of the algorithms stabilize: that of the ACORC algorithm tends toward 55%, that of QOS-POS tends toward 47%, that of MPASR is close to 40%, and that of TANARP is close to 39%.

The main reason for the behavior described above is that over time, the energies of the nodes decrease, and some nodes die prematurely because of a lack of energy, which leads to an increase in the packet loss rate. The proposed algorithm comprehensively considers the node reputation, residual energy and delay. Therefore, to a certain extent, the residual energy of each node should be similar, thereby minimizing this effect. However, the node reputation value is given priority over the other two factors, so the algorithm may choose some nodes with high reputation values but low energies. The TANARP algorithm primarily considers the node energy and does not incorporate safety factors, which is the reason why the loss rate of the proposed algorithm is slightly higher than that of the TANARP algorithm.

**Figure 10:**
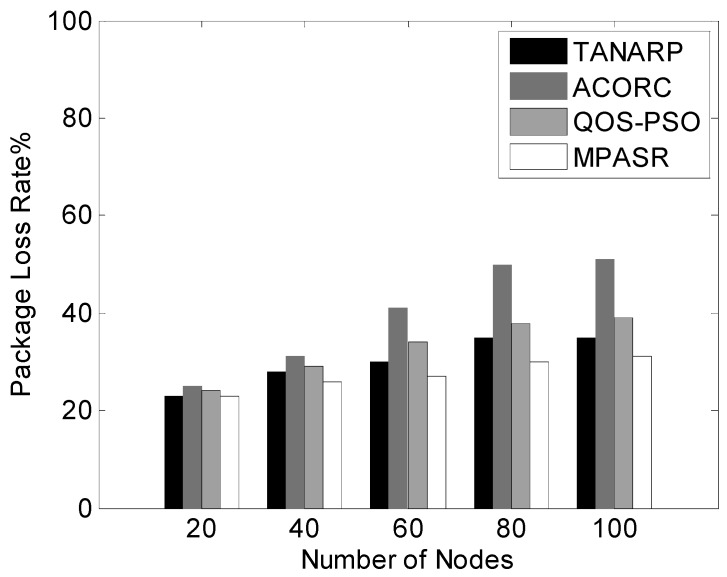
Comparison of packet loss rates for different numbers of nodes.

The data packet loss rates of the four algorithms for different numbers of nodes are shown in Figure 10. From this graph, we can see that when the number of nodes is small, the data packet loss rates of the three algorithms are not very large, but as the number of network nodes increases, the communication traffic will surge.

In this case, because network congestion results in a significant increase in the number of packets lost, the data packet loss rate of the ACORC algorithm is significantly greater than those of the TANARP, QOS-PSO and MPASR algorithms. When the number of nodes reaches 100, the packet loss rate of ACORC is 51%, that of QOS-PSO is 37%, and that of TANARP is 35%, whereas that of MPASR is only 31%. For a fixed number of malicious nodes, as the total number of nodes increases, the proposed algorithm can still maintain a low packet loss rate; this finding confirms the feasibility of this algorithm.

### 5.3. Scenario Three

In this scenario, 100 nodes of a wireless sensor network were randomly distributed in a square area of 100 m × 100 m. The proposed algorithm, the TANARP algorithm, the ACORC algorithm and the QOS-PSO algorithm are compared under the same experimental conditions. In this experiment, a fixed number of packets are sent to the sink node using a fixed data source node. The source node and sink node are located at both ends of the network. In this simulation network, including the normal nodes and malicious nodes, the number of malicious nodes accounted for fifteen percent of the total number of network nodes. We set up a number of nodes with different residual energy, which is randomly distributed between the data source and destination. These nodes are randomly deployed in the network for many times. We choose the most representative of the 10 simulation instances for comparison. Each time, the proposed algorithm, TANARP, ACORC and QOS-PSO are used to select the transmission path to send 1000 data packets from the data source to the sink node. According to the number of data packets received by the sink node, the performance of the protocols can be compared.

**Figure 11:**
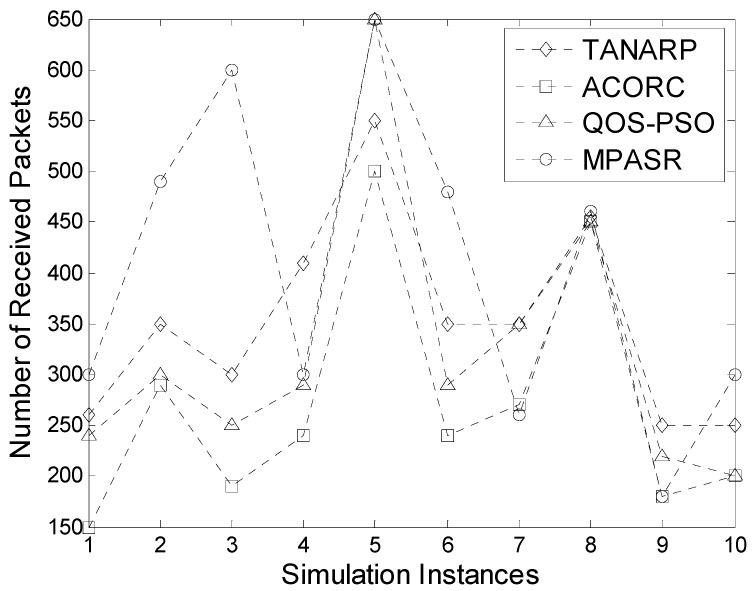
Comparison of the numbers of data packets received.

Figure 11 compares the final numbers of data packets successfully sent from the source node to the receiving node in 10 simulation instances. It can be seen from the graph that for most of the 10 instances, the MPASR algorithm resulted in the largest number of successfully sent data packets, compared with relatively small numbers for the other three algorithms. However, in the seventh simulation, the proposed algorithm yielded results similar to those of ACORC, and the results for both algorithms were inferior to those of the QOS-PSO and TANARP algorithms. The main reason for this phenomenon is that the proposed algorithm comprehensively considers the node reputation, residual energy and time delay in the pheromone calculation process, with the node reputation having the highest weight, and consequently, the algorithm will give priority to the node with the highest reputation value during data packet transmission. Therefore, it is possible that the node with the highest reputation value will be chosen as the next-hop node even if its remaining energy is very low and its time delay is relatively long. The other two algorithms are more likely to pass data packets to malicious nodes with high remaining energies and short delays. As a result, in the seventh simulation, the numbers of packets received when these algorithms were used were much higher than that achieved with the algorithm proposed in this paper. However, this is merely an isolated phenomenon. On average, the number of packets received using the proposed algorithm is higher, and it can be seen from the results that this algorithm demonstrates good performance and offers improved security of data transmission.

## 6. Conclusions

To improve the security and reliability of wireless sensor network nodes, this paper introduces a multi-attribute pheromone ant secure routing algorithm based on reputation value (MPASR). In this algorithm, ant-colony optimization is applied in the routing protocol, and the node reputation, residual energy and transmission delay are all treated as major components of the pheromone used in the optimization. In the calculation of the node reputation values, the randomness of the node distributions in wireless sensor networks is considered. Because the calculation of the reputation values may otherwise incur excessive energy consumption, we filter out unnecessary nodes by calculating the coincidence rates among the nodes. This reduces the information coincidence rate while considering network security, thereby allowing the energy consumption of the network to be reduced as much as possible to prolong the network lifetime. To ensure that the nodes can effectively resist attack from malicious nodes, the node reputation value is given the highest weight during node selection. Finally, experimental simulations are presented to compare the performances of the proposed algorithm, the TANARP algorithm, the ACORC algorithm and the QOS-PSO algorithm in terms of the packet delivery ratio, the packet loss rate, and the final number of data packets successfully received. The results fully demonstrate the superiority of the algorithm proposed in this paper.

Although this algorithm represents a considerable improvement in terms of security, it also has some shortcomings. For example, in the simulation experiment, all routing algorithms do not consider the redundant path and only select one path to transmit data. However, in practice, it is very common to use redundant paths to improve the reliability of data transmission in wireless sensor networks. In the future work, the redundant path should be taken into account. Secondly, the algorithm needs to release the query ant to judge the direction of the path in the process of routing optimization and increase the additional computing time to evaluate the attributes of the path, which has a certain effect on delay. On top of these, this paper considered only malicious nodes to simulate an attack on a wireless sensor network; however, because of the complexity of real-world networks, the ability of the experimental simulations to represent practical situations is limited. Therefore, more different attacks should be studied in the future work.

## Figures and Tables

**Table 1:** List of experimental parameters.

Parameter	Value
Number of nodes	100
Network area	100 m × 100 m
Data packet size	64 bytes
Link bandwidth	1 Mbps
Maximum data transfer rate	128 kbps
Communication range	30 m
Normal node reputation value	0.8
Malicious node reputation value	0.3
Node coincidence threshold	1.75
Weakening factor	0.4
Node group radius	10 m

**Table 2:** The number of redundant nodes under different parameter settings.

Node Coincidence Threshold	5 m	10 m	20 m
1	0	0	0
1.5	2	2	0
1.75	2	5	0
2	2	13	0
2.5	2	18	0
